# Current and Future Spatiotemporal Patterns of Lyme Disease Reporting in the Northeastern United States

**DOI:** 10.1001/jamanetworkopen.2020.0319

**Published:** 2020-03-03

**Authors:** Donal Bisanzio, Maria P. Fernández, Elisa Martello, Richard Reithinger, Maria A. Diuk-Wasser

**Affiliations:** 1 Global Health Division, International Development Group, RTI International, Washington, District of Columbia; 2 Division of Epidemiology and Public Health, School of Medicine, University of Nottingham, Nottingham, United Kingdom; 3 Earth Institute, Columbia University, New York, New York; 4 Department of Ecology, Evolution and Environmental Biology, Columbia University, New York, New York

## Abstract

**Importance:**

The incidence and geographic range of Lyme disease continues to increase in the United States because of the expansion of *Ixodes scapularis*, the species of tick that is the main Lyme disease vector. Currently, no dynamic model for the disease spread exists that integrates information of both acarological and human case surveillance data.

**Objective:**

To characterize the spatiotemporal spread of Lyme disease in humans among counties in US endemic regions.

**Design, Setting, and Participants:**

This cross-sectional study modeled the spread of Lyme disease county-level case reporting, accounting for county-level demographic factors, environmental factors associated with tick presence and human exposure, and the spatiotemporal association between counties. The analyses were conducted between January and August 2019. The setting was 1405 counties in the following regions of the United States: West North Central, East North Central, New England, Middle Atlantic, and the South. Assessments were based on publicly available Lyme disease case data reported to the US Centers for Disease Control and Prevention (CDC) between January 2000 and December 2017.

**Main Outcomes and Measures:**

Probability of reporting the first case of Lyme disease by county by year.

**Results:**

Between 2000 and 2017, a total of 497 569 Lyme disease cases were reported to the CDC in the study area. Reporting a first case of Lyme disease was associated with a county’s and county’s neighbors’ forest coverage, elevation, percentage of population living in the wildland-urban interface, tick presence, county’s population size, proportion of neighbors reporting cases, and neighbors’ years since first reporting. The model that included these variables showed high predictive power, with a mean area under the receiver operating characteristic curve of 81.1 (95% CI, 68.5-86.2). The model predicted the first reported Lyme disease case a mean (SD) of 5.5 (3.5) years earlier than was reported to the CDC, with a mean spread velocity estimated at 27.4 (95% CI, 13.6-54.4) km per year. Among 162 counties without reported cases, 47 (29.0%) had a high probability of reporting Lyme disease by 2018. The estimated mean time lag between the first reported case in a neighboring county and any county was 7 (95% CI, 3-8) years.

**Conclusions and Relevance:**

This study’s findings suggest that, if updated regularly and expanded geographically, this predictive model could enable states and counties to develop more specific Lyme disease prevention and control plans, including improved sensitization of the general population and medical community.

## Key Points

**Question:**

What are the characteristics of the spatiotemporal spread of Lyme disease in humans across counties in US endemic regions?

**Findings:**

Among 1405 counties with 497 569 Lyme disease cases in this cross-sectional study, the probability of a county reporting its first Lyme disease case between 2000 and 2017 was associated with the county’s and the county’s neighbors’ environmental and demographic factors, tick presence, and neighbors’ reporting status.

**Meaning:**

This study’s findings suggest that model results could allow states and counties to develop more specific Lyme disease prevention and control plans and strategies, including optimizing active surveillance of disease cases and ticks to increase early case detection.

## Teaser

This cross-sectional study characterizes the spatiotemporal spread of Lyme disease in humans among counties in US endemic regions.

## Introduction

Lyme disease is a multisystem zoonotic, vector-borne bacterial infection caused in North America by *Borrelia burgdorferi* sensu stricto that is transmitted by the bite of infected *Ixodes scapularis* or *Ixodes pacificus* ticks.^1,2^ *Borrelia burgdorferi* is maintained in nature in an enzootic cycle comprising multiple vertebrate species; humans are incidentally infected when exposed to infected ticks in forested or highly vegetated areas.^1,3^ With approximately 30 000 reported cases and an estimated 300 000 cases occurring anually,^4^ *B burgdorferi* is the most commonly reported vector-borne disease in the United States, representing 62.6% of cumulative reported vector-borne disease cases.^5^ Most Lyme disease cases occur in the northeastern and North Central states,^6^ where the number of endemic and high-incidence counties continues to expand.^7,8^

Quantifying the spatiotemporal spread in humans of tick-borne diseases maintained in enzootic transmission cycles remains a major challenge. Although the distribution of infected tick-borne disease vectors has been used to map the entomological or acarological risk of Lyme disease,^9,10^ vector-based data sets are generally not available longitudinally (with some exceptions, including New York State^11^) and thus are not useful to model disease spread. Furthermore, if the outcome of interest is the geographic distribution or the incidence of human disease, vector data only partially correlate with disease cases.^9,12^

The patterns of Lyme disease expansion at the national level can be inferred using US Centers for Disease Control and Prevention (CDC)–compiled reported cases based on the standardized case definition approved by the Council of State and Territorial Epidemiologists.^13^ Surveillance for Lyme disease in the United States is based on reports submitted by laboratories and health care providers to state and local health departments.^14^ As stated by Nelson et al,^4^ “These reports provide valuable insight into the age and sex distribution of patients with [Lyme disease] and the seasonality and geographic distribution of cases, and they enable monitoring of disease trends over time.”^4^^(p1625)^ Unfortunately, because of biases in diagnosis and reporting, as well as variation in surveillance practices by administrative jurisdictions, reported cases capture only a fraction of the overall frequency of Lyme disease within a population.^4^

Although both overreporting and underreporting occur,^3,15,16^ previous studies^4,17,18^ at the state and national level indicate that underreporting of clinically diagnosed Lyme disease is the dominant net outcome. However, Lyme disease underreporting is not uniform. According to Nelson et al,^4^ although 96.3% of Lyme disease cases reported to the CDC between 2005 and 2010 were concentrated in the 15 highest-incidence states, 19.4% of all clinically diagnosed cases investigated occurred outside of those states, indicating that they were missed in CDC reports. Multiple factors likely lead to increased reporting in high-incidence states, including greater public and health care provider awareness. Furthermore, the fact that a history of exposure in a state or county with at least 2 reported human cases is one of the criteria for considering a Lyme disease case as confirmed may intensify this bias and result in increased underreporting in counties or states where Lyme disease is emerging. A skewed emphasis of surveillance efforts on these high-incidence states and counties may then limit the ability to identify areas of Lyme disease emergence, where misdiagnosis or late diagnosis can lead to more severe disease manifestations.^19^

Because no standardized nationwide tick surveillance system currently exists in the United States, there is a need to maximize information extraction from data reported by the CDC in an attempt to overcome potential biases and produce actionable public health information. Previous models of reported Lyme disease spatiotemporal patterns have been generated for New York State,^18,20^ the Northeast,^8,18^ and areas undergoing geographic expansion of the disease, such as Michigan,^21^ Virginia,^22^ Minnesota,^23^ Iowa,^24^ and Pennsylvania.^19^ Our study expands the geographic scope of this work to include all of the main endemic areas in the United States identified by Kugeler et al^7^ as high-incidence states, as well as surrounding states. In addition, a novel modeling approach is presented to describe the progression of Lyme disease at a county level by estimating the yearly probability of reporting the first case of Lyme disease in each county in the study area. This approach goes beyond describing spatiotemporal patterns of cases to focus on identifying emerging areas based on ecological factors (eg, distribution of tick vectors)^8^ and human behavioral factors (eg, travel history and information sharing between neighboring counties). Specifically, a time series of case-reporting data in focal and neighboring counties was constructed between January 2000 and December 2017, as well as environmental and available tick data mechanistically linked to Lyme disease risk, to estimate associations with the probability of reporting the first case of Lyme disease in disease-free counties. The analyses were conducted between January and August 2019. This approach involved a statistical regression model, the results of which were included in a diffusion model to simulate the spatiotemporal dynamics of the probability of reporting the first Lyme disease case between 2000 and 2017. By incorporating covariates associated with the underlying ecological processes contributing to the disease spread of counties and their neighbors, it was expected that our model approach would identify nonreporting counties with high probability to begin reporting Lyme disease cases in the future.

The objectives of this study were (1) to characterize biotic and abiotic factors that altered the diffusion of Lyme disease reporting between 2000 and 2017 and (2) to identify Lyme disease–free counties with high reporting probability. Our findings can help public health authorities focus surveillance efforts in counties identified in the model as being at high risk of Lyme disease emergence but where cases have yet to be reported.

## Methods

### Study Setting and Data

This cross-sectional study included 31 states in the following regions of the United States: West North Central, East North Central, New England, Middle Atlantic, and the South^25^ (eFigure 1 in the Supplement). These regions were chosen because they include counties with a high incidence of Lyme disease,^7^ counties that started reporting cases since 2000, and counties that have never reported a Lyme disease case. Because the study involved the analysis of preexisting, deidentified data, it was exempt from institutional review board approval by RTI International and Columbia University. This study followed the Strengthening the Reporting of Observational Studies in Epidemiology (STROBE) reporting guideline.^26^

### Lyme Disease Cases

Our study used publicly available Lyme disease case data from the CDC.^27^ The reported number of cases at the county level corresponds to the cases reported from state and local health officials to the CDC through the National Notifiable Diseases Surveillance System. Cases are reported according to standardized case definitions. Starting in 2008, the case definition (note that the case definition changed again in 2011 and 2017) of Lyme disease includes probable cases (ie, a case clinically diagnosed with laboratory evidence of infection) and confirmed cases (ie, erythema migrans [the so-called bull’s-eye rash] with a known exposure or with laboratory evidence of infection or any case with ≥1 late manifestation of Lyme disease symptoms and laboratory evidence of infection).^13^ Exposure is defined as having visited a suitable tick habitat in an endemic county (or in an endemic state after the 2017 change in case definition) 30 days before onset of symptoms. A county (or a state after 2017) is considered endemic when at least 2 cases (10 cases for a state after 2017) of Lyme disease have been reported or there is an established population of infected *Ixodes* ticks. The data set used in this study included all Lyme disease cases reported between 2000 and 2017 in our study area. Therefore, the reported cases included in this study followed the case definitions established before 2017. The publicly available data from the CDC are deidentified in compliance with the Health Insurance Portability and Accountability Act Privacy Rule.

### Geographic Database

Counties’ boundaries and population estimates between 2000 and 2017 were obtained from the US Census Bureau.^25^ Forest coverage at the county level was calculated using land cover information obtained from the Multi-Resolution Land Characteristics Consortium.^28^ Total hectares of territory covered by deciduous, evergreen, and mixed forest were calculated for each county. The fraction of the population living at a wildland-urban interface (WUI) was obtained from the SILVIS Lab website.^29^ NASA Shuttle Radar Topography Mission satellite images were downloaded from the NASA website^30^ to calculate the mean elevation of each county. Information about the presence of *I scapularis* vectors in the study area was extracted from work by Eisen et al.^31^ For each county, a dichotomous variable was created in the geographic database that indicated whether a county had an established tick population or not (either occasional tick reporting or no reporting at all) as of 2015. However, those data were only available for 2000 to 2015, and the 2016 and 2017 estimates include vector data fixed at 2015 (meaning that for 2016 and 2017 the same values as for 2015 were used). All geography data were stored in a geographic database created using the software programs QGIS (version 3.4; OSGeo)^32^ and GRASS (version 7.2; OSGeo).^33^

### Statistical Analysis

A 2-step modeling approach was performed to (1) identify the relevant explanatory variables of Lyme disease reporting and the magnitude of their associations and (2) use those findings to simulate the spread of Lyme disease across the study area between 2000 and 2017, assuming a diffusionlike process. The first step consisted of a logistic regression based on generalized linear mixed models to evaluate the probability of reporting the first case of Lyme disease between 2000 and 2015, as described in the next sentence below, according to the following characteristics: a county’s population size and tick presence^31^; the percentage of the county covered by deciduous forest, evergreen forest, and mixed forest; the fraction of the population living in WUI areas; and the county’s mean elevation. For each county, the regression model also accounted for the characteristics of contiguous counties (ie, first-degree neighbors) and contiguous counties to the first-degree neighbors (ie, second-degree neighbors) to allow for potential exposure to infected vectors in out-of-county locations and the spread of infected vectors into the focal county after a diffusionlike process. The state and year were included in the model as random effects to take into account reporting variations between states and years. The regression model was performed using data between 2000 and 2015 (as training data), and Lyme disease cases reported in 2016 and 2017 were used to validate and test the model predictions. Model selection based on the Akaike information criterion was performed to identify the best model.^34^ The sensitivity and specificity of the model were estimated by calculating the area under the receiver operating characteristic curve (AUC)^35^ using the model predictions and the cases reported in 2016 and 2017. A complete description of the logistic regression model and model selection approach is included in the eAppendix in the Supplement.

The second step of the modeling approach consisted of a stochastic, spatially explicit diffusion model to predict the spatiotemporal progression of Lyme disease reporting in the study area between 2000 and 2017. This model consisted of a planar network in which each node was a county connected with the contiguous neighboring counties (eFigure 2 in the Supplement), and the diffusion of Lyme disease reporting among the counties was assessed by the coefficients obtained from the regression model. The state of Lyme disease reporting in 2000 was used as the baseline, and 1000 simulations were run for 2000 to 2017, randomly removing 100 counties in each simulation to incorporate stochasticity into the model and to reduce reporting bias. At 1-year steps, the model predicted the reporting status of each county based on the characteristics of the county and its contiguous counties. A detailed description of the diffusion model is included in the eAppendix in the Supplement. The outcomes of this model were the probability of reporting at least 1 case of Lyme disease in a county by 2018 (the predicted year for the first case being reported) and the velocity of the spread of Lyme disease. The results of the diffusion model were then compared with the observed case data obtained from the CDC. A detailed description of the method used to calculate the velocity of the spread of Lyme disease is included in the eAppendix in the Supplement.

The modeling approach accounted for the changes in case definition made by the CDC in 2008 and 2011. To avoid mixing cases reported using different case definitions, the modeling code at the beginning of each simulation randomly used the subset of reported cases in the following time windows: 2000 to 2007, 2008 to 2010, and 2011 to 2015.

## Results

Between 2000 and 2017, a total of 497 569 Lyme disease cases were reported to the CDC in the 1405 counties included in our analyses in the following regions of the United States: 27 963 in West North Central, 39 774 in East North Central, 130 992 in New England, 247 029 in Middle Atlantic, and 51 811 in the South. The publicly available data do not include sex, age, or other demographic information.

**Figure 1.**
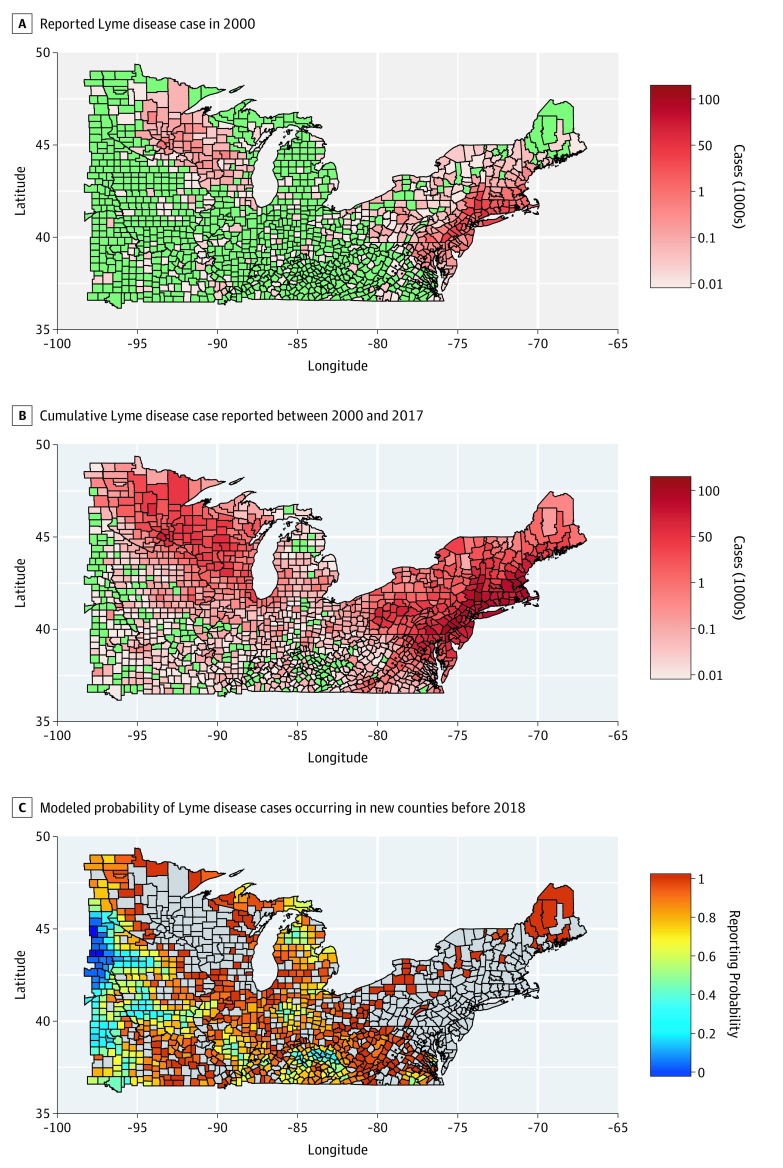
Reported Lyme Disease Cases and Estimated Probability of Case Reporting by 2018 The spread of reported Lyme disease cases predicted by the diffusion model showed high similarity to the observed data reported to the US Centers for Disease Control and Prevention.

The model selection showed that the best logistic regression model to predict the occurrence of a first case of Lyme disease between 2000 and 2017 (Figure 1) was associated with increasing county’s tick establishment (any type), decreasing elevation, increasing percentage population living in a WUI, tick presence, increasing population size, increasing proportion of first-degree neighbors reporting Lyme disease cases and tick presence since 2000, increasing year lag between the first reporting by any first-degree neighboring county, and increasing first-degree neighbors’ forest coverage (Table). The final formula of the best model is reported in the eAppendix in the Supplement. The model that included these variables showed strong classification performance (ie, predictive power), with a mean AUC of 0.81 (95% CI, 0.69-0.86). The performance of the model was substantially reduced when only county factors were considered (AUC, 0.66; 95% CI, 0.55-0.70) or when the model also included second-degree neighbors’ characteristics (AUC, 0.76; 95% CI, 0.64-0.80).

**Table.** Model Ranking Based on Results From Model Selection^a^

Characteristic	Model^b^					
	1	2	3	4	5	6
County deciduous forest coverage, hectares	/	/	/	+	+	+
County nondeciduous forest coverage, hectares	/	/	/	+	/	/
County any forest coverage, hectares	+	+	+	/	/	/
NB mean deciduous wood coverage, hectares	+	+	+	/	/	+
County elevation	−	−	−	−	+	+
NB mean nondeciduous forest coverage, hectares	−	+	+	/	/	/
County population size	+	+	+	+	+	+
NB any forest coverage, hectares	+	+	/	/	/	/
NB elevation	/	/	/	+	+	+
No. of reporting NBs	+	+	/	/	/	/
Time lag from first reporting NB	+	+	/	/	+	/
County % population living in WUI	+	/	+	+	/	+
NB mean % population living in WUI	/	+	/	/	+	/
County vector established	+	+	+	+	+	+
No. of NBs with established vector	+	+	+	/	+	/
Frequency of being selected as best model	0.81	0.11	0.05	0.04	0.01	0.01

Abbreviations: NB, first-degree neighboring county; WUI, wildland-urban interface; +, positive association; −, negative association; /, not included in the model.

^a^ Model ranking is based on the model’s descending frequency of being flagged as the best model in 1000 repetitions. Each model included the state and year as random effects to account for differences in reporting at the state level, as well as changing Lyme disease case definition between 2000 and 2015. Each model also included the population size to adjust for the increased probability to record a case of Lyme disease in counties with high populations.

^b^ A positive association means that with increasing values the greater the probability to report a Lyme case for the first time. A negative association means that with decreasing values the greater the probability to report a Lyme case for the first time.

**Figure 2.**
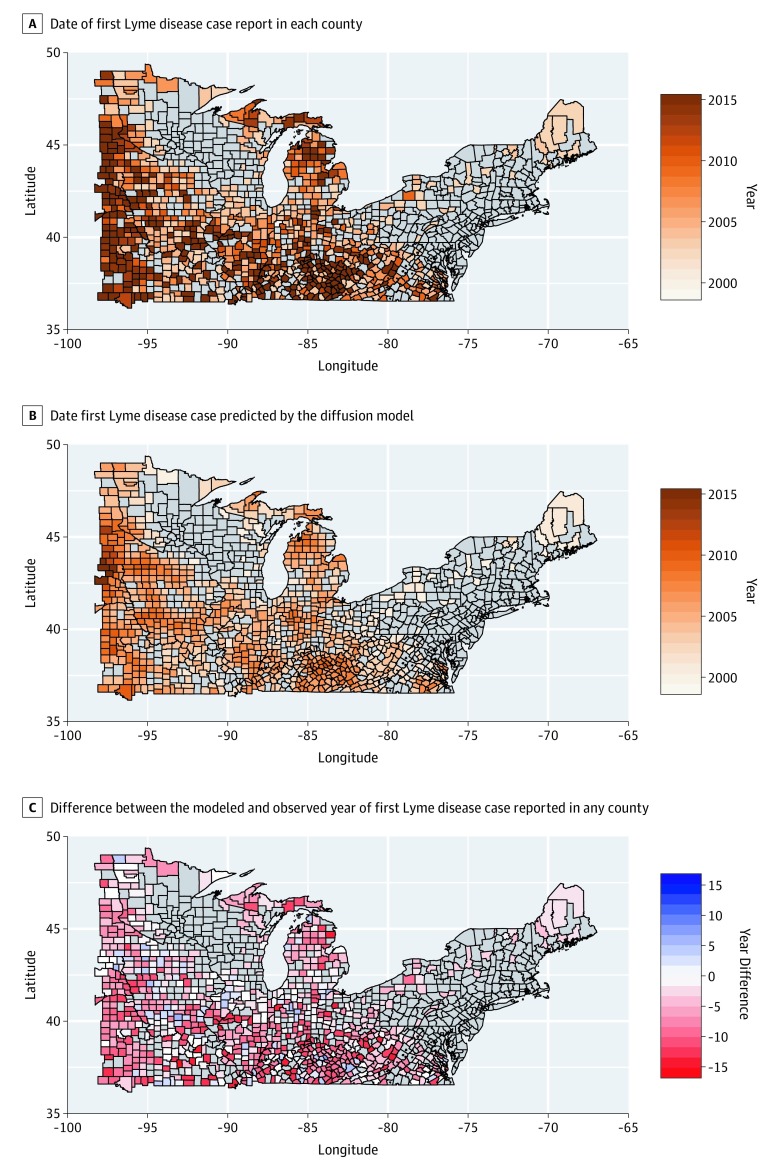
First Year of Lyme Disease Case Reporting and Modeled Spread of Lyme Disease Timing of first Lyme disease reporting estimated with the model showed a spread pattern similar to that of data reported to the US Centers for Disease Control and Prevention.

**Figure 3.**
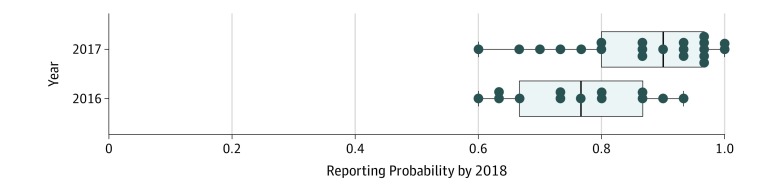
Estimated Probabilities of Counties to Report a Lyme Disease Case by 2018 if They Had Reported Lyme Disease Cases for the First Time in 2016 and 2017 The error bars represent ±1.5 times the interquartile range.

The spread of reported Lyme disease cases predicted by the diffusion model showed high similarity to the observed data reported to the CDC (Figure 2). Between 2000 and 2017, Lyme disease mostly spread toward the west and south of the study area, as well as north into Michigan’s Lower Peninsula (Figure 2A). Counties that reported cases continuously during the study period had an estimated reporting probability above 0.8 according to the diffusion model. Moreover, counties that first reported a Lyme disease case in 2016 and 2017 (data not included in the logistic regression model) showed a high reporting probability according to the model’s predictions (Figure 3). However, a mismatch was observed between the model predictions and counties not reporting Lyme disease cases between 2000 and 2017 (n = 162): 47 (29.0%) of these counties had a high (>0.8) probability of reporting Lyme disease cases by 2018 (eFigure 3 in the Supplement). This fraction varied among regions, ranging from 20.7% in West North Central to 39.4% in East North Central to 34.6% in the South.

**Figure 4.**
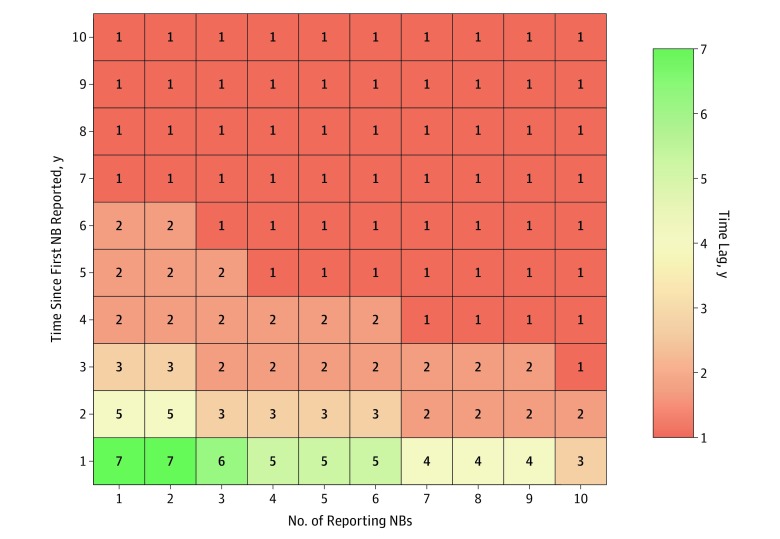
Mean Time Lag of Any County to Report a First Case of Lyme Disease Based on the Reporting Status of Neighboring Counties The number on the y-axis represents the mean year lag since the first case was reported in neighboring counties, and the number on the x-axis represents the number of reporting neighboring counties. NB indicates first-degree neighboring county.

Timing of first Lyme disease reporting estimated with the model showed a spread pattern similar to that of reported data (Figure 1A and B and Figure 2A and B). Yet, the model estimated a more rapid temporal diffusion, with the first reported Lyme disease case occurring a mean (SD) of 5.5 (3.5) years earlier than the observed data (Figure 2C). The estimated mean time lag between the first reported Lyme disease case in a first-degree neighboring county and any county was 7 (95% CI, 3-8) years (Figure 4). However, this lag decreased as the number of reporting first-degree neighboring counties increased (Figure 4). The mean Lyme disease spread velocity (as based on case reporting) was estimated at 27.4 (95% CI, 13.6-54.4) km per year for the entire study area. Diffusion velocity was higher among counties in the South (49.1 km per year; 95% CI, 25.1-70.1 km per year) compared with East North Central (21.1 km per year; 95% CI, 15.3-32.1 km per year) and West North Central (12.2 km per year; 95% CI, 6.4-21.4 km per year) counties.

## Discussion

The novel modeling approach used in this study was able to recreate the spatiotemporal pattern of Lyme disease case reporting between 2000 and 2017 by integrating Lyme disease cases reported to the national surveillance system with ecological, environmental, and spatiotemporal covariates. Our findings highlight that forest coverage, elevation, and percentage population living in a WUI are important risk factors for the spread of Lyme disease in the United States. Although such factors were linked to the occurrence of Lyme disease in previous studies reviewed by Killilea et al,^3^ our study also shows the importance of environmental characteristics of neighboring counties and their reporting status in predicting the probability of reporting Lyme disease cases. The high contribution of neighboring counties to the reporting probability may be associated with the following 3 processes acting at regional and local levels: (1) diffusion determined mainly by the introduction of the vector and the agent in a suitable habitat; (2) human-driven processes on a local scale, such as tick exposure in neighboring counties; and (3) information sharing across neighboring counties that would increase public and health care provider awareness.

The model estimated that the first report of Lyme disease cases in a county occurred on average 5.5 years earlier than was reported to the CDC, and 29.0% of counties not reporting a case between 2000 and 2017 had an 80% probability of reporting a case by 2018. We hypothesize that this earlier prediction of occurrence by our model could be associated with underreporting, including case misclassification.^18^ Such misclassification could include patients not seen with erythema migrans, the cutaneous manifestation of Lyme disease that typically prompts health care providers to inquire about travel history and request laboratory testing.^17^ Patients without erythema migrans but with other common noncutaneous manifestations of Lyme disease (eg, chronic arthritis, facial palsy, and cardiac abnormalities) should only undergo laboratory testing for Lyme disease if no other explanation for the symptoms can be found.^13^ However, health care providers working in counties where no Lyme disease cases have been reported before may not consider Lyme disease when performing differential diagnoses.^15^

Our model approach included ecological and environmental factors associated with the distribution of tick vectors and human exposure^8,36^ that would predict the occurrence of cases before they are diagnosed and subsequently reported. In emerging areas having limited experience with Lyme disease, underreporting is likely to occur more often because of diagnostic uncertainty or lack of knowledge about reporting requirements.^17^ Therefore, the model allowed us to identify potential areas where underreporting might be occurring. Once Lyme disease reporting is established in a county, then our model accurately reflects the spatiotemporal pattern observed in the cases reported to the CDC (ie, counties that first reported a Lyme disease case in 2016 and 2017 showed a high reporting probability according to the model predictions).

Our study identified that about one-third of the counties not reporting Lyme disease by 2017 had a high probability of reporting cases by 2018. This finding offers an opportunity for public health officials to anticipate and explore geographic areas where Lyme disease may be occurring or could emerge in the future. This model may be updated yearly as more cases are reported to the CDC, and the results could be used to direct active surveillance efforts to explore underreporting issues, for example.^17^ Moreover, combining case reporting with tick surveillance would offer insights into local *B burgdorferi* transmission.^37^ Our approach could help public health institutions prioritize counties for active tick surveillance or promote passive tick reporting among the general public. As tick data are collected, they may be used to expand the model and iteratively improve the model predictions.

Some differences were observed between regions regarding the diffusion dynamics of Lyme disease reporting, even after accounting for potential reporting biases among states. Although almost all counties located in the Northeast had a high probability of reporting a case between 2000 and 2017, the fraction of counties with high probability of reporting but with no observed cases by 2017 differed among the remaining regions: it was higher in the counties located in the South and the East North Central region compared with the West North Central region. Likewise, the spread velocity estimated by the model was faster in the South and the East North Central region and slower in the West North Central region. These results highlight the importance of the South and the East North Central region as areas of active spread of Lyme disease, at least at the county level, a finding that would benefit from further research to assess underlying explanatory factors.

### Limitations

This study has several limitations given the nature of the reported data that were publicly available and used in our analyses. Changes in case definitions between periods and in reporting practices across jurisdictions were accounted for by including the year and the state as covariates, but we cannot rule out additional differences in reporting practices on other levels (ie, county and local health departments). Also, Lyme disease cases are reported in the county where they are diagnosed but not necessarily where exposure to ticks occurred. Although the factors of neighboring counties were accounted for, travel history beyond first-degree and second-degree neighboring counties could also result in tick exposure and subsequent disease. Such travel could have skewed the estimated associations of the ecological, environmental, and demographic covariates included in the statistical model, particularly given that the outcome variable of the statistical model was reporting at least 1 case of Lyme disease. This limitation was overcome by performing the model selection process 1000 times and randomly excluding 100 counties from the data set in each run (eAppendix in the Supplement). Therefore, we can assume that Lyme disease cases acquired via travel would have similar associations across the study area if they occurred randomly across the regions. Our findings showed no notable association between noncontiguous counties and the occurrence of at least 1 Lyme disease case. However, we cannot rule out that exposures may have differed during travel in certain regions.

## Conclusions

Our predictive model, particularly if it is updated annually and expanded geographically, can enable states and counties to develop more specific Lyme disease prevention and control strategies, including optimizing active surveillance of human disease and ticks to increase early detection. Furthermore, this model will facilitate more targeted communication to and sensitization of the general population and the medical community to the risk of tick exposure and Lyme disease. Our findings can help public health authorities focus surveillance efforts in counties identified as being at high risk of Lyme disease emergence but where cases have yet to be reported.

## Notes

Supplement. **eAppendix.** Technical Appendix **eFigure 1.** Regional Subdivision of the Counties in the Study Area **eFigure 2.** Planar Network Used in the Stochastic Model **eFigure 3.** Estimated Probabilities of Counties to Report a Lyme Disease Case by 2018 Based on Their Status of Lyme Disease Case Reporting During the 2000-2017 Study Period, by Region. **eReferences.**
