# Supplementary material for: Current and Future Spatiotemporal Patterns of Lyme Disease Reporting in the Northeastern United States
Source: JAMA Netw Open. 2020 Mar 3;3(3):e200319. doi: 10.1001/jamanetworkopen.2020.0319 (PMC7054839; doi:10.1001/jamanetworkopen.2020.0319)
Supplement: Supplement. — eAppendix. Technical Appendix eFigure 1. Regional Subdivision of the Counties in the Study Area eFigure 2. Planar Network Used in the Stochastic Model eFigure 3. Estimated Probabilities of Counties to Report a Lyme Disease Case by 2018 Based on Their Status of Lyme Disease Case Reporting During the 2000-2017 Study Period, by Region. eReferences. [file jamanetwopen-3-e200319-s001.pdf]

Supplementary Online Content

Bisanzio D, Fernández MP, Martello E, Reithinger R, Diuk-Wasser MA. Current and future spatiotemporal patterns of Lyme disease reporting in the northeastern United States. *JAMA Netw Open*. 2020;3(3):e200319. doi:10.1001/jamanetworkopen.2020.0319

eAppendix. Technical Appendix

eFigure 1. Regional Subdivision of the Counties in the Study Area

eFigure 2. Planar Network Used in the Stochastic Model

eFigure 3. Estimated Probabilities of Counties to Report a Lyme Disease Case by 2018 Based on Their Status of Lyme Disease Case Reporting During the 2000-2017 Study Period, by Region.

eReferences

This supplementary material has been provided by the authors to give readers additional information about their work.

© 2020 Bisanzio D et al. *JAMA Network Open*.

eAppendix. TECHNICAL APPENDIX

Statistical modeling

A logistic regression analysis was performed to evaluate the effect of county characteristics on the probability to report the first LD case in a county that has never reported cases before.

Full model:

First LD case (1,0)= DEC_FOREST_{CT}+ $NON\_DEC\_FOREST_{CT}$+ANY_FOREST_{CT} + $ELEV_{CT}$ + POP_{CT} + WUI_{CT} + $VECTOR_{CT}$ + DEC_FOREST_{NB}+ $NON\_DEC\_FOREST_{NB}$+ANY_FOREST_{NB} + $ELEV_{NB}$ + WUI_{NB} + $VECTOR_{NB}$ + LD_Rep_{NB} + $YEAR\_Rep_{NB}$ + $STATE_{rnd}$ + $YEAR_{RND}$

The model included as covariates: the area covered by deciduous and non-deciduous forests (DEC_FOREST_{CT}, $NON\_DEC\_FOREST_{CT}$) and their combined coverage in the focal county (ANY_FOREST_{CT}), the county's mean elevation ($ELEV_{CT}$), and population (POP_{CT}).

Cases can also be linked to outdoor activities performed in habitats that are outside the county in which the cases are resident. To take into account this possibility, the model also included environmental characteristics of a county's neighboring counties (forest type and coverage [DEC_FOREST_{NB}+ $NON\_DEC\_FOREST_{NB}$+ANY_FOREST_{NB}] and their mean elevation [$ELEV_{NB}$]). The model's variable LD_Rep_{NB} represents the number of neighboring counties that have already reported LD cases. $YEAR\_Rep_{NB}$ is the year lag between the first reported LD case in one of the neighboring counties and the first reported LD case in the focal county. The state and year were included in the model as random effects ($STATE_{RND}$, $YEAR_{RND}$) to take into account reporting variation due to state characteristics not captured by the model's variables. The model used a binomial likelihood. The regression model was performed using a structure (STAR) model using a Markov random fields to account for spatial autocorrelation of the counties. The structure of network model used in this project is described in Bisanzio et al.[1]

Model selection approach

A multi-model selection approach based on Akaike Information Criteria (AIC) was performed to find the best models to predict occurrence of the first case reporting of LD in any given county.[2] The ΔAIC was calculated among all proposed models as the difference between their AIC and the one with the lowest AIC value. All those models showing a ΔAIC <2 were included in the set of best models.[2] To reduce bias due to reporting system in each county, model selection was performed 1,000 times and 100 counties were randomly selected and excluded by the training dataset in each run. The model with the highest frequency of being identified as the best model among the 1,000 repetitions was selected to be used in the diffusion simulator model.

Diffusion simulation

A diffusion simulator was built in order to simulate the spatio-temporal diffusion of LD reporting in the counties included in the study area from 2000–2017. The model was built to quantify the probability of each county to report their first LD cases by 2017. The simulator used a stochastic approach based on 1,000 simulations. In each simulation run, spread of LD reporting was simulated using the results of the best logistic regression model, selected as described above (eTable 1):

First LD case (1,0)= ANY_FOREST_{CT} + $ELEV_{CT}$ + POP_{CT} + WUI_{CT} + $VECTOR_{CT}$ +ANY_FOREST_{NB} + $VECTOR_{NB}$ + LD_Rep_{NB} + $YEAR\_Rep_{NB}$

© 2020 Bisanzio D et al. *JAMA Network Open*.

In order to reduce bias due to different reporting systems, in each simulation 100 counties were randomly selected and taken out from the training dataset. The logistic regression was used to estimate the probability of reporting the first case of LD in a county per each year. The prediction did only take into account the fixed effects of the logistic model. The random effects were included in the logistic regression to take into account state variability to report LD cases and changes in definition of LD cases. Thus, the probability estimation did not include the random effect to avoid the reporting bias at state level.

Each simulation run started with the Lyme disease reporting in the year 2000 and simulated the spread of cases during the following 17 years. The diffusion simulator structure was created to represent the spatial relationship among counties, with spatial proximity of counties represented in a planar network (eFigure 2). The nodes in the network are located at the counties' centroids and each node has connecting links to its first-degree neighbors. Each node was characterized by county forest coverage (combined coverage of deciduous and non-deciduous forests), mean elevation, reported first LD case (dichotomous variable), WUI, population, and time since first case reported. A single run of the simulator had 17 cycles, one per each year from 2001–2017. Per each cycle (year), the simulator calculated the probability (P_{LD}) of each county without initial cases to report a LD case, based on the parameters from the logistic regression, the county's characteristics and those of the contiguous counties. The prediction used the characteristics of the first-degree neighboring counties flagged as reporting during the previous cycles. After a P_{LD} was assigned to a county, a random number (*RN*) was extracted from a uniform distribution over the interval [0, 1] per each county. If *RN* was lower than the predicted P_{LD}, the county was set as reporting LD cases. When a county started to report cases, it remained in this status until the end of the permutation. For each county, we recorded how many times it was flagged as reporting LD cases in each of the 1,000 simulator runs. The final probability of a county to report LD from 2000 to 2017 was calculated by dividing the times the county was set as reporting cases during the whole simulation by 1,000 (i.e., the number of simulations). The 95% confidence interval (CI) was calculated using a binomial function.

Estimation of Lyme disease spread velocity

The velocity of LD spread reporting through the study area was calculated using the results of the diffusion simulator. Per each simulation, we calculated the distance from each county that reported a LD case for the first time during the last cycle of the simulation (i.e. the 17th cycle) and the closest counties that were already reporting LD in 2000. The calculated distance was divided by the number of cycles (17) to calculate the mean annual speed (km) in each run. The final diffusion speed was calculated by performing the mean of the 100 runs. Bootstrapping approach was used to calculate the 95% CI. The mean diffusion speed was calculated in each region by averaging all speeds calculated from the last reporting counties to the nearest counties reporting LD in the year 2000.

© 2020 Bisanzio D et al. *JAMA Network Open*.

eFigure 1. Regional Subdivision of the Counties in the Study Area

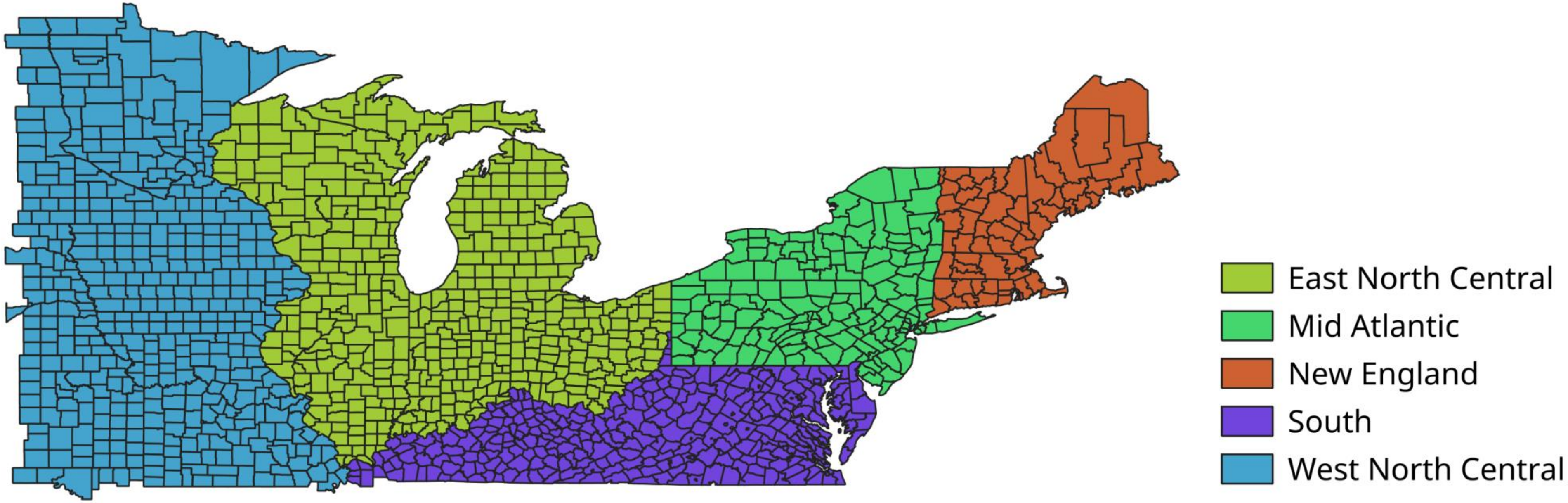

© 2020 Bisanzio D et al. *JAMA Network Open*.

eFigure 2. Planar Network Used in the Stochastic Model. Each node in the network represents the centroid of a county and each link connects nodes based on county contiguity.

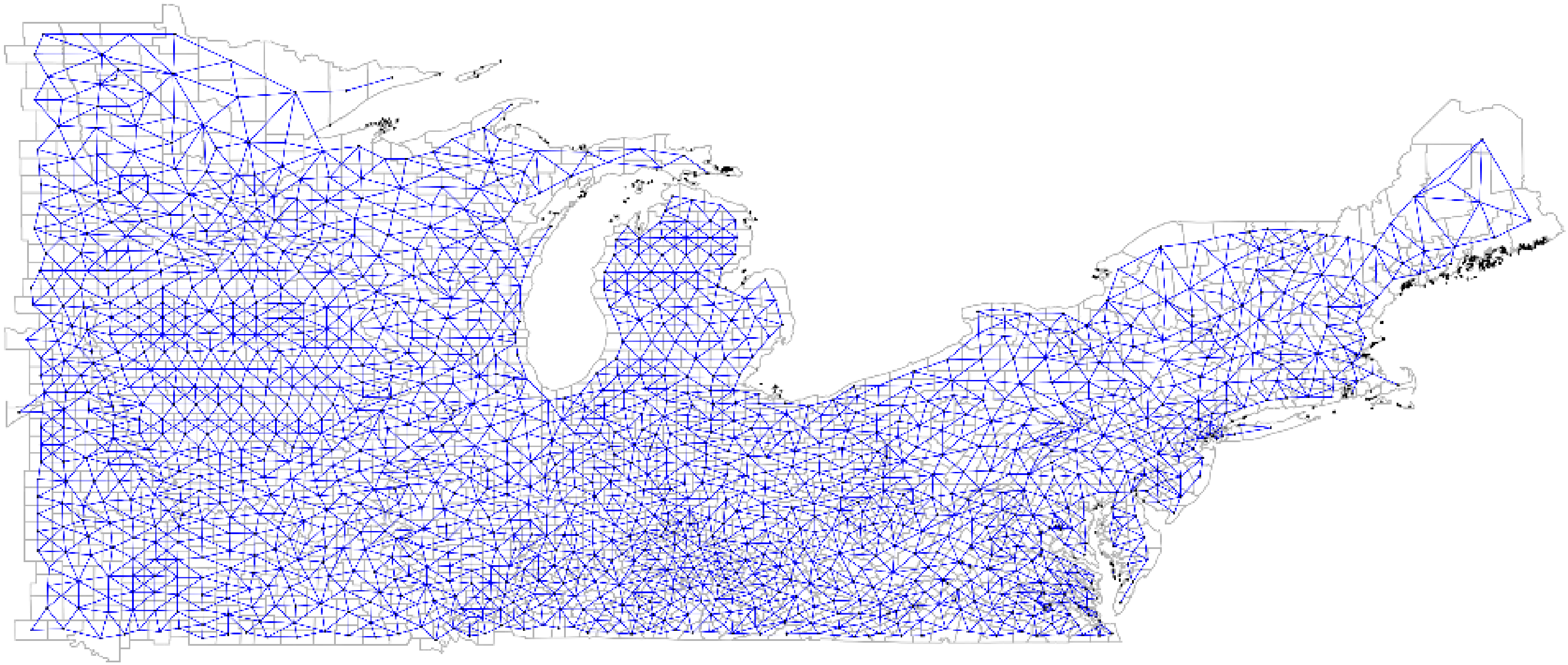

© 2020 Bisanzio D et al. *JAMA Network Open*.

eFigure 3. Estimated Probabilities of Counties to Report a Lyme Disease Case by 2018 Based on Their Status of Lyme Disease Case Reporting During the 2000-2017 Study Period, by Region. The boxplots show the estimated probability of reporting a case of LD by 2018 grouped by the mean time (in years) passed between years during which cases were reported for 2000–2017 study period.

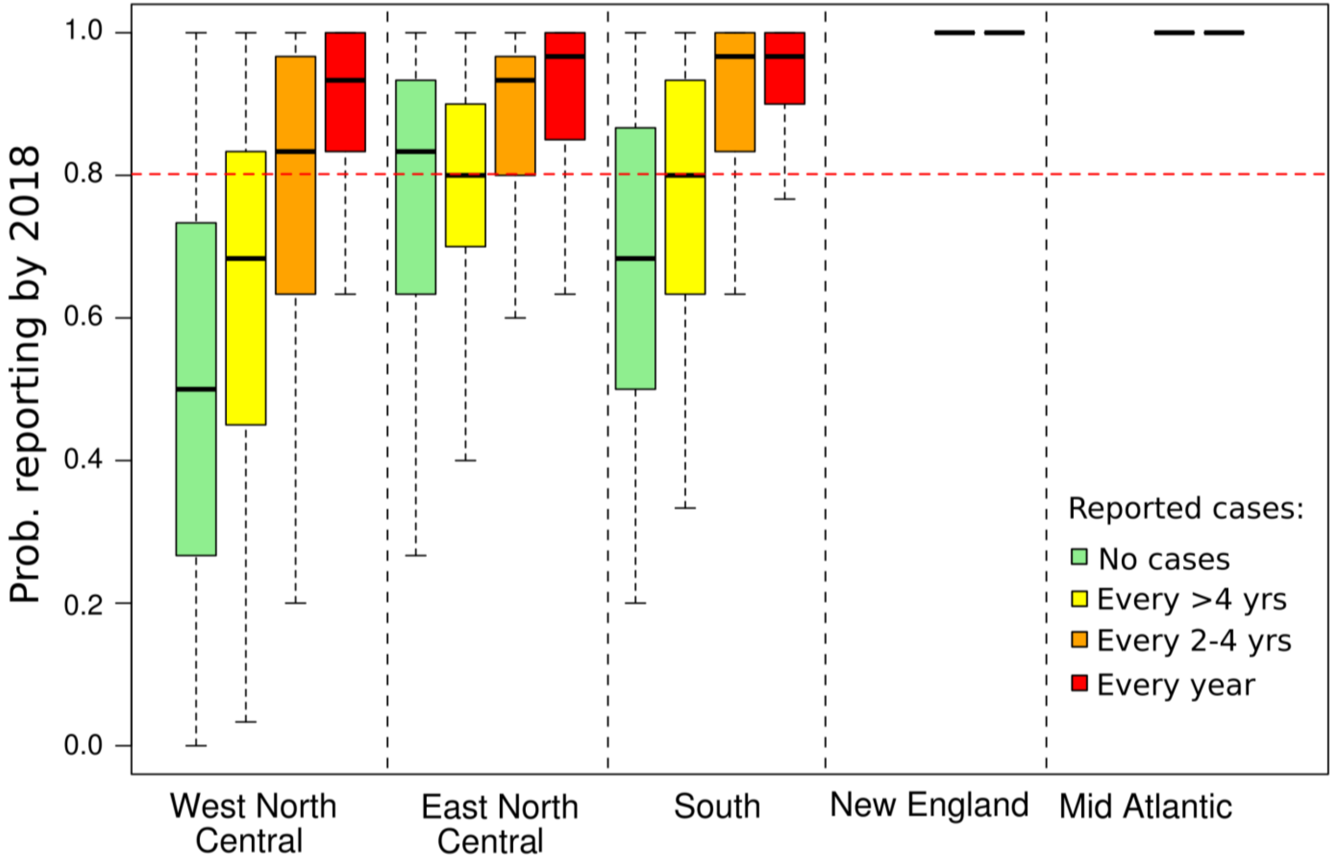

© 2020 Bisanzio D et al. *JAMA Network Open*.

eReferences

1. Bisanzio D, Mutuku F, LaBeaud AD, Mungai PL, Muinde J, Busaidy H, Mukoko D, King CH, Kitron U. "Use of prospective hospital surveillance data to define spatiotemporal heterogeneity of malaria risk in coastal Kenya." Malaria journal 14, no. 1 (2015): 482.
2. Burnham KP, Anderson DR. *Model selection and multimodel inference: a practical information-theoretic approach.* New York City, NY: Springer-Verlag; 2002.

© 2020 Bisanzio D et al. *JAMA Network Open*.
